# Term Neonatal Complications During the Second Localized COVID-19 Lockdown and Prolonged Premature Rupture of Membranes at Home Among Nulliparas With Reference Interval for Maternal C-Reactive Protein: A Retrospective Cohort Study

**DOI:** 10.3389/fped.2022.787947

**Published:** 2022-04-08

**Authors:** Yang Geng, Weihua Zhao, Wenlan Liu, Jie Tang, Hui Zhang, Weilin Ke, Runsi Yao, Ji Xu, Qing Lin, Yun Li, Jianlin Huang

**Affiliations:** Department of Obstetrics and Gynecology and Center for Perinatal Medical Health, The First Affiliated Hospital of Shenzhen University, Shenzhen Second People's Hospital, Shenzhen, China

**Keywords:** COVID-19, premature rupture of membranes, neonatology, expectant treatment, neonatal intensive care unit

## Abstract

**Objective:**

The COVID-19 lockdown extended premature rupture of membranes (PROM) expectant time among nulliparas and increased the risk of term neonatal complications. This study investigated the impact of term nulliparas with PROM delays at home on neonatal outcomes during the COVID-19 lockdown period, considering the clinical diagnostic application of maternal C-reactive protein (CRP).

**Methods:**

This study collected 505 term nulliparous women who underwent PROM at home from five provinces in a non-designated hospital of China in 2020. We analyzed PROM maternal information at home and neonatal complications in the COVID-19 regional lockdown and compared related information in the national lockdown. Poisson regression models estimated the correlation of PROM management at home, maternal CRP, and neonatal morbidity. We constructed two diagnostic models: the CRP univariate model, and an assessed cut-off value of CRP in the combined model (CRP with PROM waiting time at home).

**Results:**

In the regional lockdown, PROM latency at home and the severity of neonatal complications were extended and increased lower than in the nationwide lockdown, but term neonatal morbidity was not reduced in the COVID-19 localized lockdown. Prolonged waiting time at home (≥8.17 h) was associated with increasing maternal CRP values and neonatal morbidity (adjusted risk ratio 2.53, 95% CI, 1.43 to 4.50, *p* for trend <0.001) in the regional lockdown period. In the combined model, CRP ≥7 mg/L with PROM latency ≥8.17 h at home showed higher diagnostic sensitivity and AUC than only CRP for initial assessing the risk of adverse neonatal complications in COVID-19 regional lockdowns (AUC, 0.714 vs. 0.534; sensitivity, 0.631 vs. 0.156).

**Conclusion:**

The impact of the acute COVID-19 national blockade on the PROM newborns' health could continue to the COVID-19 easing period. Maternal CRP reference interval (≥7 mg/L) would effectively assess the risk of term neonatal morbidity when nulliparas underwent prolonged PROM expectant at home (≥8.17 h) during the second COVID-19 lockdown.

## Introduction

More than 207 million COVID-19 confirmed cases had been reported globally, with ~4.4 million deaths as of August 16, 2021, by WHO official declaration (1). After the first wave of the COVID-19 pandemic with national lockdown (January 2020–May 2020) in China, other small waves of infection happened irregularly in Beijing, Hebei, Liaoning, Guangdong, Xinjiang, and Changchun provinces with regional lockdown (June 2020–December 2020). The case fatality rate (CFR) was 15% during the early COVID-19 pandemic in Wuhan and subsequently decreased to 1.4% (2, 3). To overcome the severe shortage of available medical resources, the government constructed or retrofitted several facilities, including Fangcang shelter hospitals and non-designated hospitals (4, 5). Due to the nationwide blockade, travel and movement restrictions will inevitably affect the number of medical visits for clinical populations with regular follow-ups. In low- and middle-income countries (LMICs), where remote consultations are less feasible, nulliparas who lack birth experience might miss antenatal care during a certain pregnancy period (6–8).

After the national blockade was lifted, small-scale outbreaks and large-scale population movements led to the repeated quarantine of pregnant women. Regional quarantine reduced pregnant women's requirement for antenatal care and increased worry about their infants' and own well-being. During the delayed phase of the COVID-19 pandemic, term nulliparas were more likely to be managed at home and had a longer duration of membrane rupture before hospitalization than multiparas. England Collaborative Group indicated that 36 to 45% of nulliparous women were required to transfer to the hospital due to secondary obstetric complications, compared with 9 to 13% of multiparous women during the COVID-19 pandemic (9). The Guttmacher Institute estimates that even a moderate decrease of 10% in pregnancy-related and neonatal health care coverage could result in an additional 28,000 maternal deaths and 1,68,000 neonatal deaths globally (10). However, no finding is investigating changes in term neonatal outcomes in subsequent COVID-19 remission periods.

Premature rupture of membranes (PROM) complicates ~5–10% of all pregnancies, 60% of which occur at term (11–13). The risks of PROM and fetal distress among pregnant women during the COVID-19 pandemic were higher than those who gave birth before the pandemic (14). Fetal infection risk increases proportionally with the time between membrane rupture and delivery (15, 16). Admissions of term neonates to the neonatal intensive care unit (NICU) and unexpected postnatal complications have been proposed as neonatal-focused quality metrics (17). However, for the remission period of the epidemic, there is no relevant study to report the association between adverse nulliparas PROM at home and term newborn morbidity.

The effectiveness and quick diagnostic indicators are essential for estimating the risk of PROM neonatal complications during the COVID-19 pandemic. One study discovered the maternal serum CRP level (≥8 mg/L) obtained up to 72 h before delivery is an independent predictor of funisitis and early-onset neonatal sepsis in women with preterm labor or preterm PROM (18). CRP in cord blood level differs between term labor and preterm PROM (19). However, there were no reports on differences in the CRP reference intervals for predicting outcomes in term neonates with PROM from preterm neonates. Maternal CRP upper reference limits have been variably implemented between 5 and 10 mg/L in China, the UK, the USA, and Australia (20). There is a lack of evidence to the association between PROM latency (between membrane rupture and delivery) at home, maternal CRP results, and term neonatal morbidity. In this study, we would present adverse expectant duration of PROM at home combined with diagnostic maternal CRP reference interval as valuable indicators to guide nulliparous women with PROM at home to reduce neonatal morbidity during the COVID-19 pandemic effectively.

## Methods

### COVID-19 Setting

In this study, Shenzhen, one of the cities with the largest floating population in China, was selected to analyze pregnancy-related health care in a large-scale non-designated hospital. In total, 1,250 PROM subjects were from 5 provinces, including Guangdong, Hunan, Hubei, Jiangxi, and Hebei. When the country was under acute national COVID-19 blockade, there were no or a few movements between Shenzhen and other provinces. After the nationwide lockdown was lifted, a proportion of women from other areas returned to Shenzhen. All nulliparas were admitted without fever (maternal body temperature <37.3°C). Nucleic acid test within 48 h was negative from January 30, 2020, in national lockdown. They had not been vaccinated before pregnancy.

### Study Design and Participant Inclusion/Exclusion

This retrospective cohort study set the acute COVID-19 period with national lockdown from January 1, 2020 to May 31, 2020. The remission period with regional lockdown was from June 1, 2020 to December 31, 2020. The onset of continuous vaginal fluid or discontinuous vaginal fluid ≥3 times at home among nulliparas without regular uterine contraction was diagnosed and confirmed PROM by pH test of vaginal fluid or ferning tests in hospital (21). The start time of PROM expectant management was recorded by obstetricians based on the admission inquiry (continuous vaginal fluid or discontinuous vaginal fluid ≥3 times at home). The pregnant women's delivery time is accurately recorded in the electronic nursing records of the delivery room. The discharge summary described the mother and child's discharge details. Postnatal neonatology consultation and newborns' hospitalization recorded adverse neonatal outcomes, such as NICU admission.

**Figure 1:**
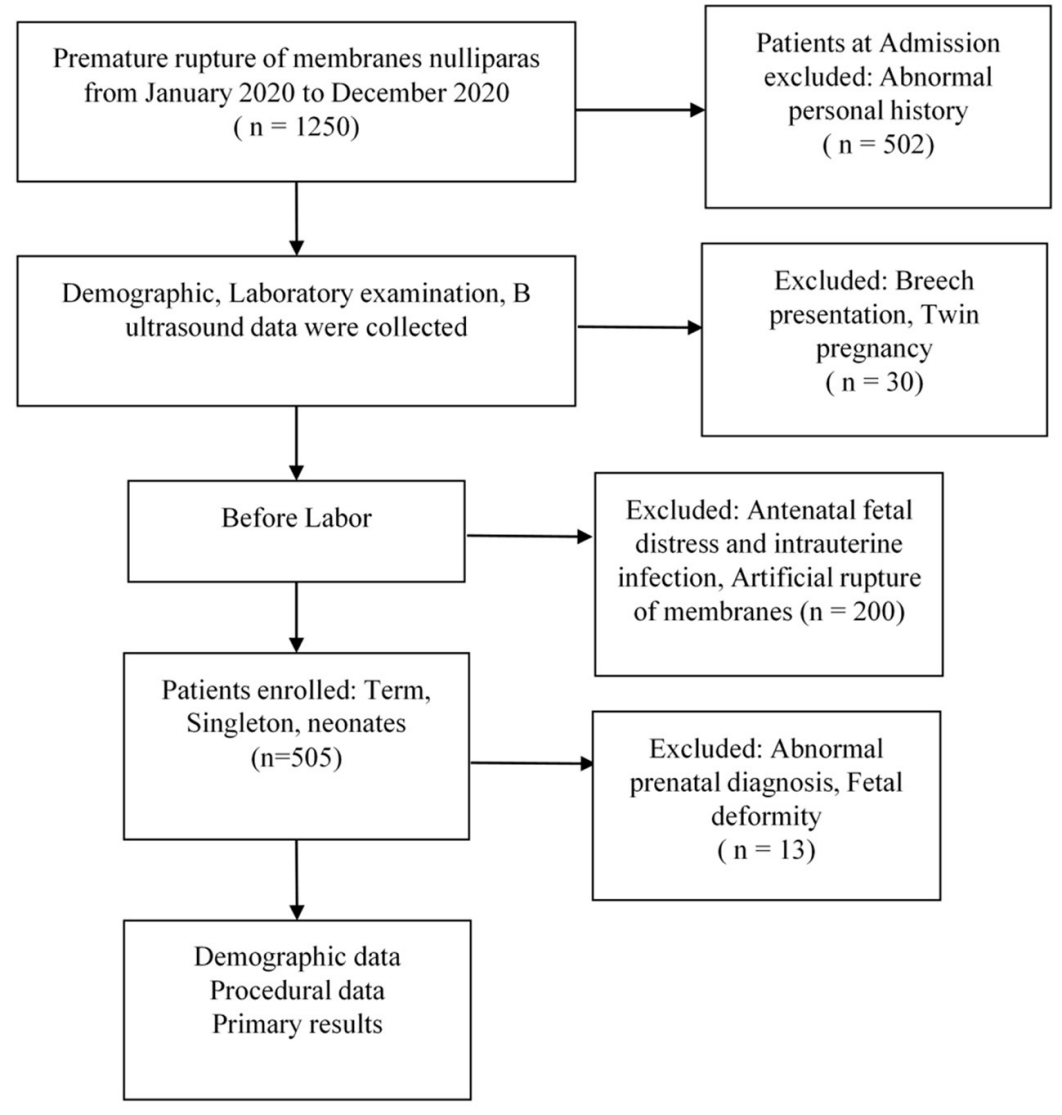
Study flow chart.

For this study, nulliparous women (age >18 and ≤40 years) who underwent PROM after gestational age ≥37 weeks with singleton and liveborn infants were included. Medical history inquired excluded adverse personal history (smoking, drug abuse, alcohol), syphilis infection, pregnancy complications (pregnant hypertension, hepatitis, systemic lupus erythematosus, diabetes mellitus), twin pregnancy, breech presentation, birth canal malformations, and maternal congenital heart disease. Before the onset of labor, PROM occurrences resulting from dilation of the cervix with Foley plus were excluded. We also excluded PROM women complicated with antenatal fetal distress, oligoamnios, and intrauterine infection. Abnormal prenatal diagnosis results were excluded, including fetal deformities and changes in the number of chromosomes. B-Ultrasound in this study included choroid plexus cysts, echogenic intracardiac focus, and mild separation of the renal pelvis in the second trimester, but the aforementioned manifestations disappeared by B-ultrasound diagnosis in the third trimester or admission day for delivery (Figure 1).

### Variables and Measurements

The primary outcomes for this study are neonatal complications, including neonates who were admitted to NICU admission, neonatal infectious pneumonia (NIP), meconium aspiration syndrome, and neonatal jaundice. Demographic variables were maternal age, gestational age, gravidity, body mass index (BMI), areas, and history of present illness (GDM, gestational diabetes mellitus; GBS, group B streptococcus infection). The most critical variable is PROM waiting time for labor at home. This continuous variable was categorized as quartiles (<4.22 h, 4.22–8.16 h, 8.17–17.14 h, >17.14 h). After admission, the PROM women were examined by blood routine, C-reactive protein (CRP), obstetric B-ultrasound, and maternal body temperature (MBT).

### Statistical Analysis

Baseline cohort characteristics were described as mean ± standard deviation (normal distribution) or median (interquartile range) (skewed distribution), and categorical variables were presented as a percentage. The *t*-test (normal distribution), non-parametric tests (skewed distribution) test, and χ^2^ tests (categorical variables) were used to determine any statistical differences between the means and proportions of the groups. One-way ANOVA and generalized linear models (Gamma distribution) detect differences at CRP in different waiting-time levels at home. *P*-value for trend was tested on the association between waiting time at home (categorical variables) and CRP (log), based on one-way ANOVA. Poisson regression models evaluated every level of PROM waiting time at home, maternal CRP, BMI, and gestational age association with neonatal morbidity.

Pearson's correlation, and correlations between CRP (Log) and PROM latency (Log) were analyzed. According to the STROBE statement's recommendation, we simultaneously showed the results of crude risk ratios (cRRs), minimally adjusted risk ratios (aRRs), and fully adjusted analyses, with 95% CI, respectively.

ROC analysis was performed to calculate the area under the curve (AUC) to evaluate the models' diagnostic performance. We computed the AUC with a 95% CI using 500 bootstrap re-sampling (22).

The relative weight for predicting neonatal incidence was determined by converting parameter estimates calculated with generalized linear models to calculate the diagnostic score. Diagnostic scores were calculated by using the following formulas:

Model I Score = β_CRP_ × Parameter _CRP_

Model II Score = β_CRP_ × Parameter _CRP_ + β_waitingtimeathome_ × Parameter _waitingtimeathome_

where β is the coefficient.

The optimal CRP cut-off value was applied to calculated AUC, specificity, and sensitivity in subgroups and both lockdowns in every model.

Analyses were performed using statistical software R (The R Foundation; http://www.r-project.org; version 3.5.1) and SPSS Statistic 26.0 and *p* < 0.05 (two-sided) were used to define statistical significance.

## Results

### Characteristics of Nulliparous Women and Adverse Neonatal Outcomes With PROM Between Two Lockdown Periods

There were 224 and 281 pregnant nulliparous women onsets of PROM in the COVID-19 national and regional lockdown periods, respectively. Gestational age, BMI, gravidity, and number of populations from five areas were comparable in both periods (Table 1). There were no differences in the proportion of GDM, GBS infection, prenatal B ultrasound, and fetal position. Nulliparous women admission ratios after PROM observed a significant increase during the day (7:00–19:00) and decline at night (20:00–6:00) in regional lockdown compared with the national lockdown (during the day, 190, 67.62% vs. 117, 52.23%, *p* = 0.001; at night, 91, 32.38% vs. 107, 47.77%, *p* = 0.001). A significant decrease in PROM duration at home was noted in the COVID-19 regional lockdown period compared with that in national periods (median, IQR: 6.18 (10.85) vs. 11.00 (14.34), *p* < 0.001) (Table 1). More PROM women extended expectant treatment at home more than 17.14 h in the national lockdown period (74/224, 33.04%), but in the regional lockdown, 35.59% (100/281) PROM women delayed expectant duration < 4.22 h at home (Table 1). Clinical examination indications of maternal CRP and MBT decreased in regional lockdown compared with national lockdown period [CRP median (IQR), 1.79 (3.92) vs. 2.68 (4.55), *p* < 0.001; MBT, means (SD), 36.48 (0.18) vs. 36.52 (0.19), *p* = 0.03]. Maternal cesarean section incidence resulting from fetal distress did not decrease in the regional lockdown period (Table 1).

**Table 1:** Characteristics of nulliparous women with PROM between COVID-19 national and regional lockdown periods.

	**COVID-19 National lockdown**	**COVID19 Regional lockdown**	***P*-value**
	**(** ***N*** **, %; mean, SD/** **median, IQR)**	**(** ***N*** **, %; mean, SD/** **median, IQR)**	
Maternal age (years)	29.3 (3.51)	28.4 (3.26)	0.005^*^
Maternal age (years)	224	281	0.005^*^
<25	12 (5.36)	31 (11.03)	
25-35	196 (87.50)	240 (85.41)	
>35	16 (7.14)	10 (3.56)	
GA (days)	273.42 (6.54)	273.77 (7.28)	0.59
GA (weeks)	224	281	0.22
37 (259–265)	36 (16.07)	44 (15.66)	
38 (266–272)	65 (29.02)	72 (25.62)	
39 (273–279)	77 (34.38)	89 (31.67)	
40 (280–286)	46 (20.54)	76 (27.05)	
Gravidity (times)	224	281	0.15
1	135 (60.27)	186 (66.19)	
2	56 (25.00)	63 (22.42)	
≥3	33 (14.73)	32 (11.39)	
BMI (kg/m^2^)	26.33 (3.12)	26.10 (2.90)	0.40
BMI (kg/m^2^)	224	281	0.59
<25	80 (35.71)	107 (38.08)	
≥25	144 (64.29)	174 (61.92)	
GDM	224	281	0.12
No	186 (83.04)	247 (87.90)	
Yes	38 (16.96)	34 (12.10)	
GBS	224	281	
No	208 (92.86)	262 (93.24)	0.87
Yes	16 (7.14)	19 (6.76)	
Bishop score			0.18
<6	157 (70.09)	212 (75.44)	
≥6	67 (29.92)	69 (24.56)	
CRP (mg/L)	2.68 (4.55)	1.79 (3.92)	<0.001^*^^a^
WBC (× 10^9^/L)	8.79 (2.66)	8.83 (3.09)	0.61 ^a^
AFI (mm)	81.70 (1.61)	81.92 (1.53)	0.92
MBT (°C)	36.52 (0.19)	36.48 (0.18)	0.03^*^
Prenatal B ultrasound	224	281	0.94
Normal	190 (84.82)	239 (85.05)	
Abnormality	34 (15.18)	42 (14.95)	
Fetal position	224	281	
Occiput position	219 (97.77)	265 (94.41)	0.05
Others	5 (2.23)	16 (5.69)	
Area	224	281	0.11
Guangdong	100 (44.64)	136 (48.40)	
Hunan	34 (15.18)	33 (11.74)	
Hubei	31 (13.84)	29 (10.32)	
Jiangxi	34 (15.18)	33 (11.74)	
Hebei	25 (11.16)	50 (17.80)	
Admission time	224	281	0.001^*^
Day	117 (52.23)	190 (67.62)	
Night	107 (47.77)	91 (32.38)	
Wait-time at home (h)	11.00 (14.34)	6.18 (10.85)	<0.001^*^^a^
Wait-time at home (h)	224	281	<0.001^*^
<4.22	27 (12.05)	100 (35.59)	
4.22–8.16	56 (25.00)	70 (24.91)	
8.17–17.14	67 (29.91)	59 (21.00)	
>17.14	74 (33.04)	52 (18.51)	
Cesarean section			0.25
No	176 (78.6%)	233 (82.9%)	
Yes	48 (21.4%)	48 (17.1%)	

*GA, gestational age; BMI, body mass index; GDM, gestational diabetes mellitus; GBS, group B streptococcus; CRP, C-reactive protein; WBC, white blood cell count; AFI, amniotic fluid index; MBT, maternal body temperature*.

a *Non-parametric tests, median, IQR*.

>* *Statistical significance*.

Neonatal Apgar score <7 at 5 min and neonatal rescue in both periods did not differ. The overall composite of adverse neonatal outcomes did not decrease in the regional lockdown period compared with national lockdowns (Table 2).

**Table 2:** Adverse term neonatal outcomes between COVID-19 national and regional lockdown periods.

**Neonatal outcomes**	**COVID-19 national lockdown**	**COVID19 national lockdown**	***p*-value**
	**(means, SD/** **median, IQR) (** ***N*** **, %)**	**(means, SD/** **median, IQR) (** ***N*** **, %)**	
Fetal weight	3,184.2 (335.2)	3,151.8 (377.7)	0.33
Apgar score <7 at 5 min			0.23
No	211 (94.20)	271 (96.44)	
Yes	13 (5.80)	10 (3.56)	
Neonatal intubation			0.29
No	204 (91.07)	263 (93.59)	
Yes	20 (8.93)	18 (6.41)	
Neonatal disease			0.38
No	118 (52.68)	159 (56.58)	
Yes	106 (47.32)	122 (43.42)	
Composite items	224	281	
NICU			0.15
No	132 (58.93)	183 (65.12)	
Yes	92 (41.07)	98 (34.88)	
NIP			0.21^a^
No	205 (91.52)	265 (94.41)	
Yes	19 (8.48)	15 (5.34)	
MAS			0.64
No	209 (93.30)	265 (94.31)	
Yes	15 (6.70)	16 (5.69)	
Neonatal jaundice			0.40
No	144 (64.29)	191 (68.97)	
Yes	80 (35.71)	90 (32.03)	

*NICU, neonatal intensive care unit; NIP, neonatal infectious pneumonia; MAS, meconium aspiration syndrome*.

a *Fisher exact test*.

### Association Between PROM Wait-Time at Home and Relative Risks of Neonatal Complications or CRP Values

After adjusted areas, maternal age and neonatal confounders like gestational age, BMI, gravidity, amniotic fluid index (AFI), GDM, GBS infection, fetal weight, and aRRs of neonatal morbidity were 6.06 (95% CI, 1.41 to 26.01, national lockdown) and 2.53 (95% CI, 1.43 to 4.50, regional lockdown) (8.17–17.14 h vs. <4.22 h). aRRs of neonatal morbidity were 9.81 (95% CI, 2.31 to 41.68, national lockdown) and 3.48 (95% CI, 1.98 to 6.11, regional lockdown) (>17.14 h vs. <4.22 h) (Table 3). The *p*-value for trend for aRRs of adverse neonatal outcomes both increased as extended at home after PROM during the COVID-19 pandemic (*p* for trend <0.001).

**Table 3:** Association between PROM wait-time at home and relative risks of neonatal complications or CRP values.

**Neonatal composite outcomes**	**COVID-19 national lockdown**			**COVID-19 regional lockdown**		
	**Model I**	**Model II**	**Model III**	**Model I**	**Model II**	**Model III**
	**cRRs**	**aRRs**	**aRRs**	**cRRs**	**aRRs**	**aRRs**
		**95% CI**			**95% CI**	
**Wait-time at home (h)**						
<4.22	1.00	1.00	1.00	1.00	1.00	1.00
4.22–8.16	1.69 (0.35, 8.12)	1.63 (0.34, 7.88)	1.40 (0.29, 6.86)	1.79 (0.99, 3.22)	1.78 (0.99, 3.22)	1.78 (0.97, 3.26)
8.17–17.14	7.05 (1.70, 29.32)^*^	7.19 (1.73, 29.95)^*^	6.06 (1.41, 26.01)^*^	3.05 (1.77, 5.27)^*^	3.01 (1.73, 5.24)^*^	2.53 (1.43, 4.50)^*^
>17.14	11.31 (2.77, 46.24)^*^	11.48 (2.79, 47.19)^*^	9.81 (2.31, 41.68)^*^	3.94 (2.31, 6.73)^*^	3.95 (2.31, 6.75)^*^	3.48 (1.98, 6.11)^*^
*P* for trend	<0.001^*^	<0.001^*^	<0.001^*^	<0.001^*^	<0.001^*^	<0.001^*^
CRP (mg/L)	COVID-19	Difference (95% CI)	*P*-value	COVID-19	Difference (95% CI)	*P*-value
	National lockdown (marginal means, SD)			Regional lockdown (marginal means, SD)		
**Wait-time at home (h)**						
<4.22	1.88 (0.36)	1.00		2.04 (0.25)	1.00	
4.22–8.16	2.49 (0.33)	1.33 (0.84, 2.10)	0.23	2.66 (0.39)	1.30 (0.89, 1.90)	0.17
8.17–17.14	3.95 (0.48)	2.10 (1.34, 3.28)	0.001^*^	3.82 (0.62)	1.87 (1.26, 2.78)	0.002^*^
>17.14	5.48 (0.64)	2.92 (1.88, 4.53)	<0.001^*^	5.92 (1.02)	2.90 (1.92, 4.39)	<0.001^*^
*P* for trend	<0.001^*^			0.04^*^		

*Model I: a univariate model without controlling for any confounding factors*.

*Model II: controls for areas and maternal age*.

*Model III: based on model II, supplemented to control gestational age, body mass index, gravidity, gestational diabetes mellitus, group B streptococcus infection, amniotic fluid index, fetal weight*.

*cRR, crude risk ratio; aRR, adjusted risk ratio*.

* *Statistical significance*.

PROM waiting time at home significantly correlated with CRP between both lockdown periods (Pearson correlation _nationallockdown_, r = 0.28, 95% CI, 0.16 to 0.40, *p* < 0.001; Pearson correlation _regionallockdown_, r = 0.17, 95% CI, 0.05 to 0.28, *p* = 0.005). Further study of the association between the PROM waiting time at home and maternal CRP values is shown in Table 3. Waiting time within 8.17–17.14 h or >17.14 h groups comparing with time intervals <4.22 h were associated with higher maternal CRP values, [national lockdown period, marginal means (3.95 vs. 1.88), difference 2.10 (95% CI, 1.34 to 3.28), *p* = 0.001] and [regional lockdown period, marginal means, (3.82 vs. 2.04), difference 1.87 (95% CI, 1.26 to 2.78), *p* = 0.002] (8.17–17.14 h vs. <4.22 h); [national lockdown period, marginal means (5.48 vs. 1.88), difference 2.92 (95% CI, 1.88 to 4.53), *p* < 0.001] and [regional lockdown period, marginal means, (5.92 vs. 2.04), difference 2.90 (95% CI, 1.92 to 4.39), *p* < 0.001] (>17.14 h vs. <4.22 h) (Table 3). *P*-value for trend indicated that the CRP values were gradually rising with extended waiting time for labor at home based on every PROM latency level during the COVID-19 pandemic (*p* for trend <0.001 in the national lockdown; *p* for trend = 0.04 in the regional lockdown) (Table 3).

### Correlation of CRP, BMI, Gestational Age With Neonatal Complications, CRP, and Neonatal Complications Under BMI and Gestational Age Subgroups

CRP as a continuous variable was associated with neonatal complications, and there were comparable aRR values in both lockdown periods, 1.03 (95% CI, 1.01 to 1.06, national lockdown) and 1.02 (95% CI, 1.01 to 1.03, regional lockdown). During COVID-19 regional lockdown, nulliparous women BMI ≥25 kg/m^2^ compared with BMI <25 kg/m^2^ had a higher risk of adverse neonatal complications, aRR, 1.36 (95% CI, 1.02 to 1.81) (Table 4).

**Table 4:** Correlation of CRP, BMI, gestational age with neonatal complications, CRP, and neonatal complications under BMI and gestational age subgroups.

**Neonatal composite outcomes**	**COVID-19 national lockdown**			**COVID-19 regional lockdown**		
	**Model I**	**Model II**	**Model III**	**Model I**	**Model II**	**Model III**
	**cRRs**	**aRRs**	**aRRs**	**cRRs**	**aRRs**	**aRRs**
		**95% CI**			**95% CI**	
**CRP (mg/L)**						
	1.03 (1.01, 1.06)^*^	1.04 (1.01, 1.07)^*^	1.03 (1.01, 1.06)^*^	1.02 (1.01, 1.02)^*^	1.02 (1.01, 1.03)^*^	1.02 (1.01, 1.03)^*^
**BMI (kg/m** ^**2**^ **)**						
<25	1.00	1.00	1.00	1.00	1.00	1.00
≥25	1.41 (1.02, 1.94)^*^	1.38 (1.01, 1.91)^*^	1.25 (0.91, 1.72)	1.22 (0.91, 1.62)	1.21 (0.90, 1.62)	1.36 (1.02, 1.81)^*^
**Gestational age (weeks)**						
40~40^+6^	1.00	1.00	1.00	1.00	1.00	1.00
37~37^+6^	1.22 (0.83, 1.80)	1.25 (0.84, 1.86)	1.14 (0.73, 1.76)	1.18 (0.82, 1.70)	1.19 (0.82, 1.73)	0.77 (0.49, 1.22)
38~38^+6^	0.83 (0.55, 1.25)	0.84 (0.56, 1.27)	0.79 (0.51, 1.22)	0.81 (0.55, 1.20)	0.82 (0.56, 1.21)	0.65 (0.43, 1.00)
39~39^+6^	0.88 (0.60, 1.30)	0.88 (0.60, 1.28)	0.91 (0.62, 1.32)	0.88 (0.62, 1.25)	0.87 (0.61, 1.23)	0.82 (0.57, 1.17)
Neonatal composite outcomes	Model I (cRR, 95% CI)	Model II (aRR, 95% CI)	Model III (aRR, 95% CI)	Model I (cRR, 95% CI)	Model II (aRR, 95% CI)	Model III (aRR, 95% CI)
**ln CRP (mg/L) (risk variable)**						
**BMI (kg/m** ^**2**^ **)**						
<25	1.07 (0.85, 1.34)	1.06 (0.84, 1.35)	0.97 (0.76, 1.24)	1.01 (0.87, 1.18)	1.01 (0.88, 1.17)	1.03 (0.90, 1.20)
≥25	1.04 (0.92, 1.17)	1.04 (0.91, 1.18)	1.03 (0.91, 1.16)	1.13 (1.02, 1.26)^*^	1.13 (1.02, 1.26)^*^	1.13 (1.02, 1.26)^*^
**Gestational age (weeks)**						
37~37^+6^	1.07 (0.88, 1.30)	1.00 (0.86, 1.18)	0.97 (0.81, 1.16)	1.03 (0.87, 1.22)	1.04 (0.87, 1.24)	1.02 (0.84, 1.23)
38~38^+6^	1.10 (0.86, 1.40)	1.03 (0.82, 1.29)	1.01 (0.83, 1.23)	1.00 (0.92, 1.22)	0.98 (0.81, 1.18)	0.98 (0.82, 1.19)
39~39^+6^	1.04 (0.84, 1.22)	1.02 (0.85, 1.23)	0.97 (0.80, 1.18)	1.07 (0.90, 1.26)	1.07 (0.90, 1.27)	1.15 (0.97, 1.36)
40~40^+6^	1.13 (0.90, 1.42)	1.11 (0.89, 1.38)	1.10 (0.89, 1.36)	1.20 (1.05, 1.38)^*^	1.21 (1.05, 1.39)^*^	1.22 (1.05, 1.40)^*^

*Mode I: a univariate model without controlling for any confounding factors*.

*Model II: controls for areas and maternal age*.

*Model III: based on model II, supplemented to control gestational age, body mass index (BMI), gravidity, gestational diabetes mellitus, group B streptococcus infection, amniotic fluid index, and fetal weight (excluded BMI and gestational age in their subgroups)*.

*cRR, crude risk ratio; aRR, adjusted risk ratio*.

* *Statistical significance*.

Subgroup analysis observed that lnCRP was associated with the increased neonatal incidence among nulliparous women BMI ≥25 kg/m^2^ subgroup during regional lockdowns, aRR 1.13 (95% CI, 1.02 to 1.26). When gestational age was 40~40^+6^ weeks, increased maternal lnCRP during regional lockdowns worsened neonatal morbidity, aRR 1.22 (95% CI, 1.05 to 1.40) (Table 4).

### Construction of Diagnostic Models

We chose CRP and PROM waiting time at home as candidate parameters and constructed two diagnostic model I and model II. Diagnostic scores can be calculated by using the following formulas:

Model I Score = 0.07 × Parameter _CRP_

Model II Score = 0.02 × Parameter _CRP_ + 0.11

× Parameter _waitingtimeathome_.

**Figure 2:**
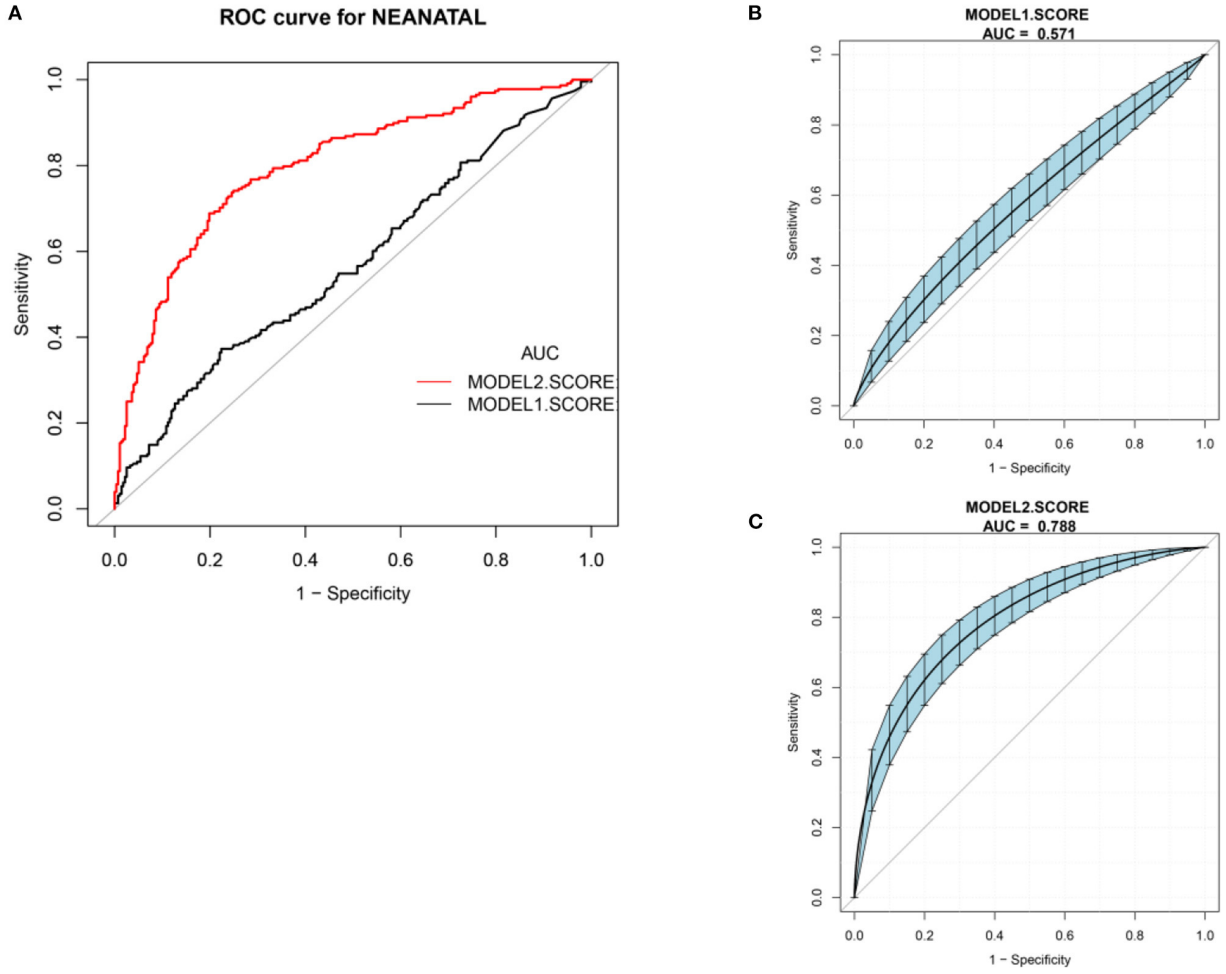
ROC curve. **(A)** Total ROC curve of model I and model II. **(B)** Model I AUC with a 95% confidence interval using 500 bootstrap re-sampling, 0.571 (95% CI, 0.523 to 0.622). **(C)** Model II AUC with a 95% confidence interval using 500 bootstrap re-sampling, 0.788 (95% CI, 0.751 to 0.825).

ROC curves for these cohorts are shown in Figure 2. The AUC of model I and model II were 0.571 (95% CI, 0.523, 0.622) and 0.788 (95% CI, 0.751. 0.825), respectively. The cut-off value of the diagnostic score at the optimum point was 1.04 in model II. Sensitivity, specificity, and negative and positive predictive values (Npv and Ppv) were 0.741, 0.751, 0.779, and 0.710. Model I's sensitivity, specificity, Npv, and Ppv were 0.373, 0.776, 0.601, and 0.578, respectively. Putting the 8.17 h of the PROM waiting time into model II got the CRP cut-off value of 7.14 mg/L.

### AUC, Sensitivity, and Specificity in Two Lockdowns and Subgroup Analysis in Regional Lockdown With CRP of 7 mg/L and PROM Latency of 8.17 h

Sensitivity and specificity were described with optional cut-off values (7 mg/L for CRP, 8.17 h for PROM waiting time at home). Model I (CRP) showed high specificity and low sensitivity in national and regional lockdowns to diagnose neonatal complications (nationwide lockdown, 0.873 and 0.198 vs. regional lockdown, 0.911 and 0.156). With model II (CRP + PROM waiting time at home) to test the diagnostic accuracy of neonatal complications, results indicated that sensitivity in both lockdowns significantly increased from 0.198 and 0.156 up to 0.915 and 0.631 (Table 5).

**Table 5:** AUC, sensitivity, and specificity in both lockdowns and subgroup analysis in regional lockdown with CRP of 7 mg/L and PROM latency of 8.17 h.

**Mode**	**Specificity**	**Sensitivity**	**Positive pv**	**Negative pv**	**AUC**
**COVID-19 national lockdown**					
Model I: CRP (≥7 mg/L)	0.873	0.198	0.583	0.548	0.536
Model II: CRP (≥7 mg/L) + WT (≥8.17 h)	0.627	0.915	0.688	0.892	0.782
**COVID-19 regional lockdown**					
Model I: CRP (≥7 mg/L)	0.911	0.156	0.576	0.585	0.534
Model II: CRP (≥7 mg/L) + WT (≥8.17 h)	0.786	0.631	0.694	0.745	0.714
**COVID-19 regional lockdown (BMI** **≥25 kg/m**^**2**^**)**					
Model II: CRP (≥7 mg/L) + WT (≥8.17 h)	0.774	0.630	0.708	0.706	0.706
**COVID-19 regional lockdown (gestational age** **=** **40~40**^**+6**^ **weeks)**					
Model II: CRP (≥7 mg/L) + WT (≥8.17 h)	0.805	0.714	0.758	0.767	0.769

*WT, PROM waiting time at home; Positive pv, positive predictive value; Negative pv, negative predictive value*.

There was little change in AUC, sensitivity, and specificity during regional lockdowns by model II in BMI ≥ 25 kg/m^2^ subgroup analysis (AUC 0.706, sensitivity 0.630, specificity 0.774). However, in the gestational age of the 40 weeks subgroups, model II improved in AUC, specificity, and sensitivity for the diagnosis of adverse neonatal complications (AUC 0.769, sensitivity 0.714, specificity 0.805).

## Discussion

This study sought to understand the impact of pregnant management at home on term newborns among PROM nulliparas during the COVID-19 regional lockdown. Our findings indicated that PROM nulliparas without regular contractions were more likely to be admitted to the hospital in the day during localized lockdown periods. PROM latency at home and the severity of neonatal complications were reduced and lessened during the second blockade period. However, neonatal morbidity in regional lockdowns was not distinguished from the acute COVID-19 phases. In subgroup analysis, according to maternal BMI ≥ 25 kg/m^2^ and 40~40^+6^ weeks of gestation in regional lockdowns, increased maternal CRP was associated with higher risks of neonatal morbidity after PROM at home. Specific CRP conference intervals (≥7 mg/L) among PROM nulliparas combined with prolonged waiting time (≥8.17 h) at home can preliminarily estimate the risk of neonatal complications.

This study consisted of outcomes in regional lockdown and national lockdown rather than in pre-COVID periods because lifestyle and medical environment changed by the pandemic need more attention and research. Kugelman et al. found a higher proportion of women with PROM in a COVID-19 cohort than pre-COVID-19 (20.6% vs. 11.0%, *p* < 0.001) (23). The most onset of term PROM has been shown to have a 24-h rhythm with peak contraction between midnight and 6:00, and timing of term PROM occurrence between midnight and 4:00 (24, 25). We observed that nulliparous women with PROM during regional lockdown were more likely to be admitted during the day than at night compared with the national lockdown period. After full lifting of China's nationwide lockdown, the large-scale population moved to various provinces and cities. Hospitals and governments issued strict pandemic prevention and control measures: people with travel histories were quarantined for 14 days in designated facilities or home settings (26). PROM nulliparous women without regular uterine contractions were more willing to wait until the daytime due to stringent measures.

In the second regional lockdown, a higher proportion of PROM patients (35.59%) stayed at home for <4.22 h, and 33.04% of PROM expectant management >17.14 h at home were in the national lockdown. Although the regional lockdown period shortened the PROM latency at home, our study indicated that neonatal complications did not differ between the acute COVID-19 phases and subsequent remission period. Lei et al. reported that quarantine people had a greater prevalence of anxiety and depression than those not affected by quarantine in southern China during 2020 (27). Adverse mental-health effects on pregnant women worsen several unfavorable pregnancy outcomes (28), leading to considerable risks for fetal complications in regional lockdowns.

It was reported that PROM women managed for longer than 24 h, and their infants tended to receive antibiotics and be in the NICU (16). In pre-COVID-19 periods, full-term nulliparas who experienced PROM without regular uterine contractions would go to the hospital for relevant medical examinations and care as soon as possible to prevent intrauterine infection and adverse neonatal complications in China. One-third of women underwent inadequate antenatal visits in the acute COVID-19 pandemic, resulting in 44.7% of pregnancies showing complications (28, 29). The present study showed that PROM latent interval at home prolonged to ≥8.17 h increased maternal CRP values and neonatal morbidity in both lockdown periods. The risk ratios of adverse neonatal outcomes in the national lockdown phase were ~2.5 times the second wave of the regional lockdown period.

The burden of obstetric and non-obstetric infections, which is particularly profound in LMICs, feasibility, standardization, and cost of time-saving are key considerations in LMICs (30, 31). Our results indicated that an increased CRP (continuous variable) aggravated the risk of neonatal morbidity in term neonates during both lockout periods, 1.03 (95% CI, 1.01 to 1.06, national lockdown) and 1.02 (95% CI, 1.01 to 1.03, regional lockdown). CRP reference intervals reported in guidelines and studies vary substantially, such as ≥5mg/L in China, ≥7 mg/L in the UK, and ≥10 mg/L in the USA and Australia (31). Using CRP (model I) for estimating PROM neonatal complications, AUC, sensitivity, and specificity were 0.571, 0.373, and 0.776, respectively. With the addition of PROM latency at home, model II improved diagnostic performance compared with CRP only: AUC, sensitivity, and specificity were 0.788, 0.741, and 0.751, respectively. The same performance was that model II (CRP ≥7 mg/L, PROM waiting time at home ≥8.17 h) showed significantly higher AUC and sensitivity than model I in two lockdown studies (regional lockdown AUC, 0.714 vs. 0.534, sensitivity, 0.631 vs. 0.156; national lockdown AUC, 0.782 vs. 0.536, sensitivity, 0.915 vs. 0.198). Model II was more favorable for nulliparas with prolonged PROM latency at home because higher sensitivity improved initial screening for neonatal complications after admission and helped obstetricians quickly estimate the risk of neonatal morbidity.

According to the WHO guidelines, obesity is considered one of the crucial risk factors predisposing to COVID-19 (32). Previous studies showed abnormal BMI of pregnant women increased the risk of gestational diabetes, significant for gestational age, and neonatal Apgar scores < 7 at 5 min (33). PROM nulliparas with a BMI ≥ 25 kg/m^2^ during the local blockade increased the rate of neonatal morbidity compared with maternal BMI < 25 kg/m^2^. In a regional lockdown, increasing CRP among overweight nulliparas (BMI ≥ 25 kg/m^2^) was associated with higher rates of neonatal complications, aRRs 1.13 (95% CI, 1.02 to 1.26).

Composite neonatal morbidity in the present study worsened as increasing CRP within 40~40^+6^ weeks of gestation, aRRs 1.22 (95% CI, 1.05 to 1.40). Even if the rate of actual morbidity is low among term uncomplicated nulliparous women (39–40 weeks of gestation), adverse neonatal outcomes in NICU, meconium aspiration syndrome, and sepsis could result from a modifiable morbid condition (34). For example, maternal psychosocial and mental health under strict quarantine measures in regional lockdowns improved in response to inflammation and manifested high concentrations of CRP (28, 35). In the present study, PROM nulliparas at 40~40^+6^ weeks of gestational age subgroup analyses observed in model II (CRP ≥7 mg/L, PROM latency at home ≥8.17 h) increased the diagnostic sensitivity for neonatal morbidity from 0.631 to 0.714, and AUC from 0.714 to 0.769 in regional lockdown periods.

### Study Limitations

The limitation of our study was that it was an observational, retrospective study conducted in a monocentric non-designated hospital over a limited period. Although PROM women were from five provinces in China, the number of subjects were indefinite about floating population during regional lockdown period. It has been reported that the concentration of CRP cannot be assumed to be stable in different periods of pregnancy (31, 36). CRP in these data was only one test result for PROM nulliparas admitted to the hospital, which may cause information bias. However, we suggested that CRP would be a valuable indicator to assess the effect of extended expectant treatment of PROM at home on adverse neonatal outcomes during the COVID-19 pandemic.

## Conclusion

This study might guide term nulliparas with PROM who inevitably extended waiting time at home during the second COVID-19 lockdown periods: When PROM latency at home was ≥8.17 h, nulliparas with maternal CRP ≥7 mg/L estimated the risk of neonatal complications effectively in this study.

## Data Availability Statement

The original contributions presented in the study are included in the article/supplementary material, further inquiries can be directed to the corresponding authors.

## Ethics Statement

The studies involving human participants were reviewed and approved by the First Affiliated Hospital approved the study protocol of Shenzhen University, the Clinical Research Ethics Committee of Shenzhen Second People's Hospital (20210713001-FS01). The patients/participants provided their written informed consent to participate in this study.

## Author Contributions

YG designed experiment investigation, methodology, software, and wrote the original draft. WZ and WL conducted the investigation, validation, project administration, software, and funding. JT, WK, RY, and HZ conducted article reviews, collected resources, and investigated. QL, JX, YL, and JH reviewed the article again and detected methodology. All authors read and approved the final article.

## Funding

The present study was supported by the National Nature Science Foundation of China (grant number 81901438) and Discipline Construction Funds for Critical Maternal Treatment (grant number 4001005).

## Conflict of Interest

The authors declare that the research was conducted in the absence of any commercial or financial relationships that could be construed as a potential conflict of interest.

## Publisher's Note

All claims expressed in this article are solely those of the authors and do not necessarily represent those of their affiliated organizations, or those of the publisher, the editors and the reviewers. Any product that may be evaluated in this article, or claim that may be made by its manufacturer, is not guaranteed or endorsed by the publisher.
